# Autonomic Nervous Dysfunction in Hamsters Infected with West Nile Virus

**DOI:** 10.1371/journal.pone.0019575

**Published:** 2011-05-09

**Authors:** Hong Wang, Venkatraman Siddharthan, Jeffery O. Hall, John D. Morrey

**Affiliations:** Institute for Antiviral Research, Department of Animal, Dairy, and Veterinary Sciences, Utah State University, Logan, Utah, United States of America; University of Hong Kong, Hong Kong

## Abstract

Clinical studies and case reports clearly document that West Nile virus (WNV) can cause respiratory and gastrointestinal (GI) complications. Other functions controlled by the autonomic nervous system may also be directly affected by WNV, such as bladder and cardiac functions. To investigate how WNV can cause autonomic dysfunctions, we focused on the cardiac and GI dysfunctions of rodents infected with WNV. Infected hamsters had distension of the stomach and intestines at day 9 after viral challenge. GI motility was detected by a dye retention assay; phenol red dye was retained more in the stomachs of infected hamsters as compared to sham-infected hamsters. The amplitudes of electromygraphs (EMGs) of intestinal muscles were significantly reduced. Myenteric neurons that innervate the intestines, in addition to neurons in the brain stem, were identified to be infected with WNV. These data suggest that infected neurons controlling autonomic function were the cause of GI dysfunction in WNV-infected hamsters. Using radiotelemetry to record electrocardiograms and to measure heart rate variability (HRV), a well-accepted readout for autonomic function, we determined that HRV and autonomic function were suppressed in WNV-infected hamsters. Cardiac histopathology was observed at day 9 only in the right atrium, which was coincident with WNV staining. A subset of WNV infected cells was identified among cells with hyperplarization-activated cyclic nucleotide-gated potassium channel 4 (HCN4) as a marker for cells in the sinoatrial (SA) and atrioventricular (AV) nodes. The unique contribution of this study is the discovery that WNV infection of hamsters can lead to autonomic dysfunction as determined by reduced HRV and reduced EMG amplitudes of the GI tract. These data may model autonomic dysfunction of the human West Nile neurological disease.

## Introduction

WNV infection can cause acute and long-term neurological sequelae with disease signs and symptoms that reflect autonomic dysfunctions. The most widely recognized WNV-induced disease sign controlled by autonomic function is respiratory distress [1], [2], which can result in respiratory failure with a poor prognosis [3]. Of 32 patients with developing paralysis and acute WNV infection, 38% developed respiratory failure [1]. However, respiratory insufficiency may not be the only autonomic dysfunction that results from WNV infection. Early in the WNV epidemic, gastrointestinal (GI) symptoms were recognized [4] and have continued to be a predominant symptom [5], [6], [7]. Of 23 WNV patients at the Cleveland Clinic, 43% had gastrointestinal complaints [6].

Cases of cardiac or kidney involvement, although much less frequent, have been reported [8]. A case study of a 68-year-old man with a confirmed case of WNV developed clinical myocarditis coincident with WNV-IgM seroconversion. The patient developed cardiac arrhythmias, global myocardial dysfunction, and elevated cardiac enzymes; the family declined postmortem examination. Cardiac complications including arrhythmia have been described in a subsequent report in hospitalized patients with WNV disease [9]. Additionally, WNV myocarditis has been reported upon necropsy [10]. There are many reports of WNV cardiac involvement in other mammalian [11] and avian species [12]. Moreover, cardiac involvement has been reported with other arbovirus infections [13], [14], [15]. In regards to urinary function, 22% of patients with WNV-induced acute flaccid paralysis developed bladder dysfunction [16]. A case study report claimed to be the first report of a urological sequelae in a patient with WNV; the patient also had respiratory distress requiring intubation [17]. Other more subtle autonomic dysfunctions may also occur in WNV neurological disease. For example, adrenal insufficiency, as detected by a corticotropin test, was identified in 7 of 10 patients with severe WNV disease [18].

Hamsters infected with WNV have modeled aspects of the human infection and neurological disease. As in human patients, WNV infects neurons in the brain [19] and motor neurons in the spinal cord to cause poliomyelitis-like disease [20]. Unlike the human disease where the mortality rate is less than 1%, the mortality rate in hamsters can vary between 20–100% depending on the age of the animals, source and strain of the virus, and route of inoculation [21]. Likewise, the onset of disease is more rapid in hamsters compared to humans; in hamsters viremia is gone by day 6, the acute neurological disease due to infection in the central nervous system (CNS) occurs between day 4 to day 18, which is followed by long-term neurological disease lasting beyond 100 days after initial viral challenge [22]. However like in humans, WN disease signs are varied between individuals [21], which is consistent with varied disease signs [21], and multifocal neurological lesions that occur in hamsters [22], [23]. Virus and histopathology can persistently infect the kidneys and CNS tissues of hamsters, as may occur in human patients [22], [24], [25]. Respiratory distress has been partially modeled in WNV-infected hamsters where the amplitudes of electromyographs (EMGs) are suppressed in the diaphragms without evidence of WNV infection or pathology of the diaphragmatic muscle fibers [23]. This suggests that WNV-induced respiratory distress has a neurological origin. This suppression at early time-points after infection was more closely associated with WNV-infected neurons located in the medulla oblongata than in the cervical or thoracic spinal cord, i.e., infected neurons were identified in the medulla, but not in the spinal cord. Later in the infection, however, infected neurons were in both anatomical locations. In light of these findings, we hypothesized that respiratory distress was probably caused by other neurological deficits than just infection of motor neurons in the spinal cord. In the current study, we demonstrated that WNV can cause dysfunction of the autonomic nervous system, as indicated specifically by cardiac and GI dysfunctions in rodents.

## Results

**Figure 1:**
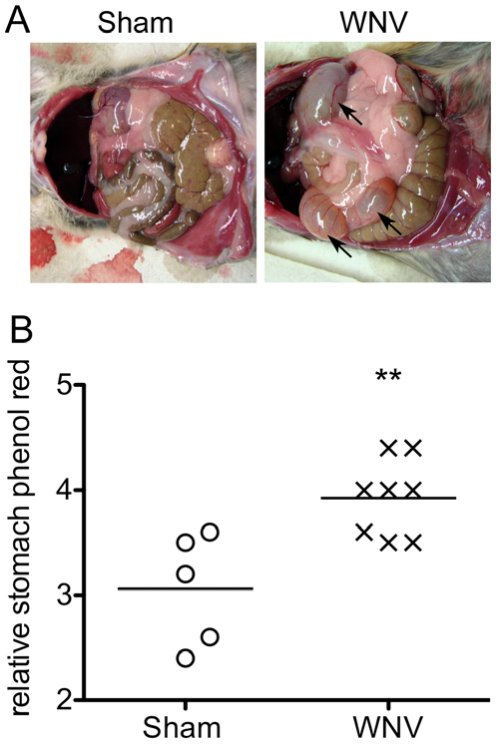
Gastrointestinal motility in WNV-infected hamsters. A) Gastrointestinal distension in WNV- or sham-infected hamsters 9 days after s.c. viral challenge (arrows). B) WNV- or sham-infected hamsters fasted for 15 hr were administered 1.5 mL of phenol red solution at 7–9 days after s.c. viral challenge. Upon necropsy, relative amounts of phenol red dye were measured in the stomachs. The higher levels of phenol red dye indicated a reduced gastrointestinal motility. (**P≤0.01 using a two-way t test.)

Due to prior observations that patients with WNV disease can exhibit respiratory distress [1], [2] and GI symptoms [4], [5], [6], [7] and prior results that WNV causes suppression of EMGs of the diaphragm of hamsters [23], we investigated the effect of WNV on other autonomic functions. Upon necropsy of WNV-infected hamsters, we observed distention of the GI tract in some of the animals at day 9 after viral challenge (Figure 1A). To investigate GI function, fasting hamsters at days 7–9 after viral challenge were orally administered phenol red dye and then necropsied 10 minutes (min) later to measure the levels of phenol red dye retained in the stomachs (Figure 1B). WNV-infected hamsters significantly (P≤0.01) retained more phenol red dye in their stomachs as compared to sham-infected hamsters.

**Figure 2:**
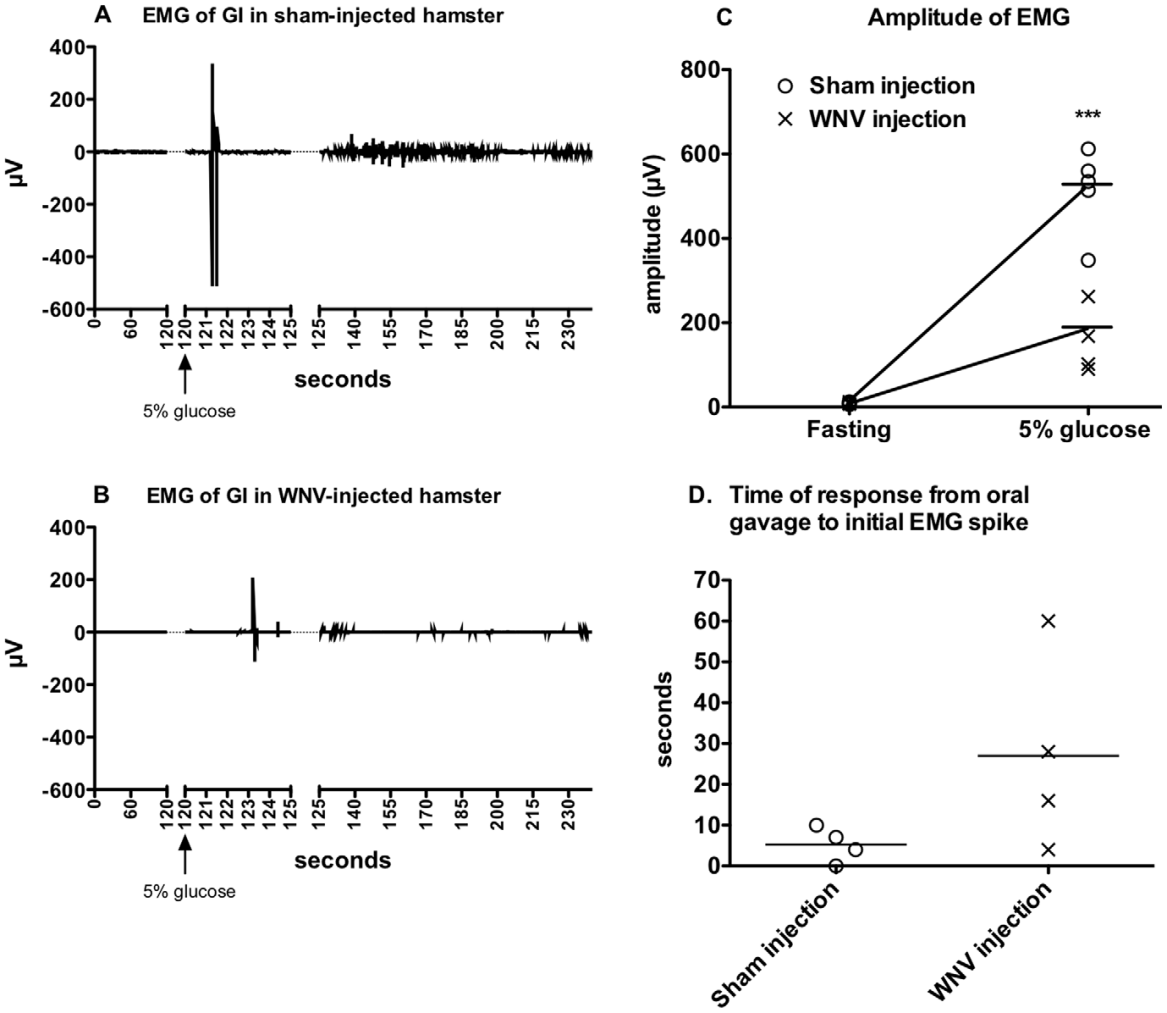
Electromyography (EMG) of intestines in WNV-infected hamsters. Hamsters were surgically implanted with telemetry transmitters with electrodes attached to the duodenum just below the pyloric sphincter, and then injected s.c with WNV or sham. At 9 days after viral challenge, EMGs were measured continuously for 4 min in isoflurane-anesthetized hamsters that had fasted for 15 hr. At 2 min (arrows), hamsters were orally gavaged with 1.5 mL of 5% glucose. Representative EMG tracings of A) sham-injected and B) WNV-injected hamsters. C) Mean amplitudes ± SD from 0–2 min. (fasting) and 2–4 min. (***P≤0.001 using a two-way t test.)

Since infection of the neurological system might electrophysiologically affect GI motility, we measured the EMG of the duodenum in isoflurane-anesthetized fasting hamsters 2 min before, and 2 min after the oral administration of 5% glucose solution (Figure 2). Examples of EMG recordings for these times are shown for a sham-infected (Figure 2A) and WNV-infected hamster (Figure 2B). The average mean ± SD of the EMG amplitudes for 2 minutes before glucose administration and for 2 minutes after glucose administration are shown (Figure 2C). The EMG amplitudes for sham-infected hamsters were significantly greater than for WNV-infected hamsters (P≤0.001). These EMG data indicated that GI muscles of WNV-infected hamsters were receiving less nerve stimulation than the sham-infected hamsters, which could account for WNV-induced GI stasis.

There was a tendency as illustrated in the two animals shown in Figure 2A and B for WNV-infected animals to have delays in the initial spikes of EMG readings in response to the glucose (all data not shown). However, the measurements in the delays of the initial EMG spikes were imprecise, because the timing of the oral gavage of the glucose administration was imprecise. Hence, these delay data were not shown.

**Figure 3:**
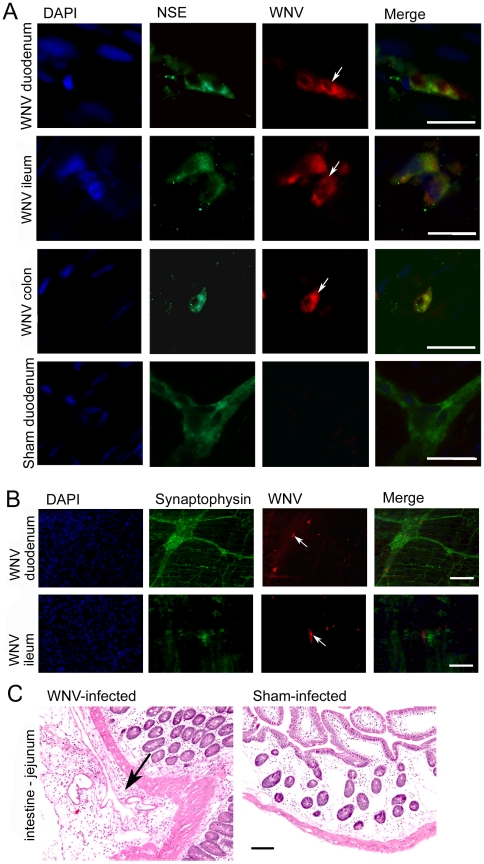
WNV-immunostaining and histopathological immunostaining of the GI tract in WNV-infected hamsters. Hamsters were injected s.c. with WNV or sham, and necropsied 9 days later. The intestine (duodenum, jejunum, ileum, colon) was sectioned for A) fluorescent immunohistochemical co-staining of WNV envelope and NSE and DAPI (nuclear), and B) WNV envelope and synaptophysin and DAPI. C) H&E staining of a representative section of intestine is shown. WNV-stained cells (white arrow). histopathological lesion (black arrow). Bar scale 100 µm.

Besides being innervated by the spinal cord and the brain stem, which are infected by WNV in humans [6], [10], [26] and hamsters [19], [20], [22], [27], [28], the GI tract is innervated by an enteric nerve complex. The myenteric plexus, between the longitudinal and circular layers of muscles in the GI tract, provides sympathetic and parasympathetic motor and secretomotor innervations. Some of these myenteric neurons were infected by WNV as evidenced by co-staining for WNV envelope (env) and NSE in the duodenum, ileum, and colon (Figure 3A). No WNV-staining was observed in sham-infected hamsters nor in sections where the primary WNV-specific antibody was removed as a control. These cells were identified to be from the myenteric plexus due to the co-staining of WNV env with synaptophysin (Figure 3B), which is present in neuroendocrine cells and in virtually all neurons in the central nervous system (CNS) involved in synaptic transmission [29]. Since we were only able to dissect and isolate only very small representative films of myenteric plexus throughout the hamster intestine due to the difficulty of the technique, we could not estimate the percentage of infected myenteric neurons; we simply knew that the virus was capable of infecting such neurons.

The peritoneal side of the intestinal omentum from all 6 of the WNV-infected hamsters, but none of the 6 sham-infected hamsters, contained histopathological lesions. Examples of these lesions were lymphocytes, plasma cells and neutrophils scattered throughout the opposing omentum resulting in localized peritonitis (Figure 3C, arrow). However, no significant inflammation was noted in the vicinity of the myenteric plexus containing infected neurons.

**Figure 4:**
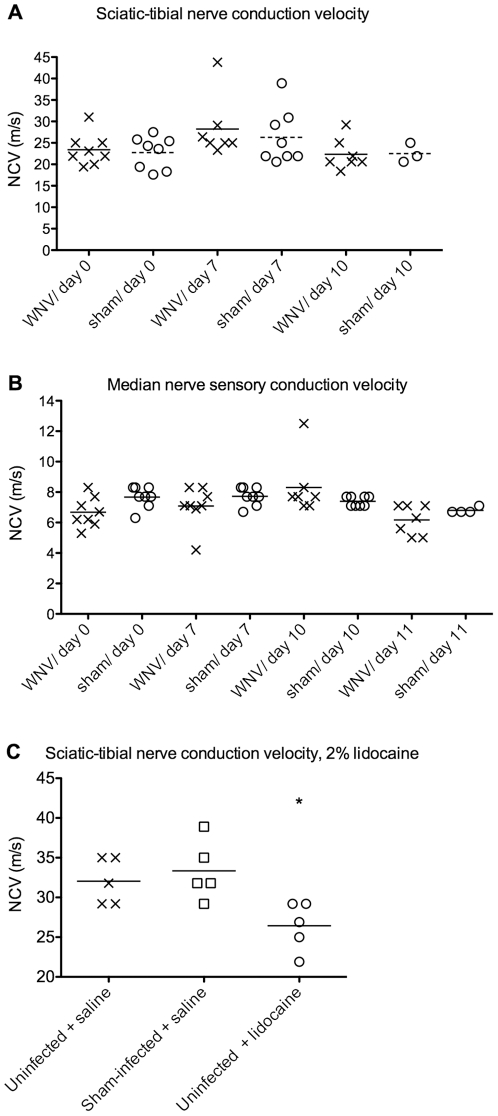
Nerve conduction velocity (NCV) in WNV- or sham-infected hamsters. Hamsters were injected s.c with WNV or sham (cell culture supernatant), and the NCV was recorded at days 0, 7, and 10 from the A) sciatic-tibial nerve, B) median sensory nerve. C) As a positive control to see if the procedure could detect differences in nerve conduction velocity, NCV was measured in uninfected or sham-infected hamsters injected i.m. with 2% lidocaine or saline in the vicinity sciatic-tibial nerve one day earlier. (*P≤0.05 using one-way analysis of variance.)

To determine if degenerated nerve fibers originating from the spinal cord caused the GI stasis or EMG suppression, sensory and motor nerve conduction velocity (NCV) studies were performed on the sciatic and median nerve in one experiment (Figure 4A and B). To determining if the NCV assay could detect defects in hamsters treated with a positive control, lidocaine, we conducted an experiment in which NCV was measured in uninfected or sham-infected hamsters (s.c.) injected i.m. one day earlier with lidocaine or saline control (Figure 4C). No statistical differences were observed in the NCV of WNV infected- compared with sham-infected animals, whereas the lidocaine suppressed NCV as predicted. Even though these nerves do not contain type B autonomic fibers, these results still suggest that nerve degeneration did not generally occur and was not likely responsible for autonomic dysfunction.

**Figure 5:**
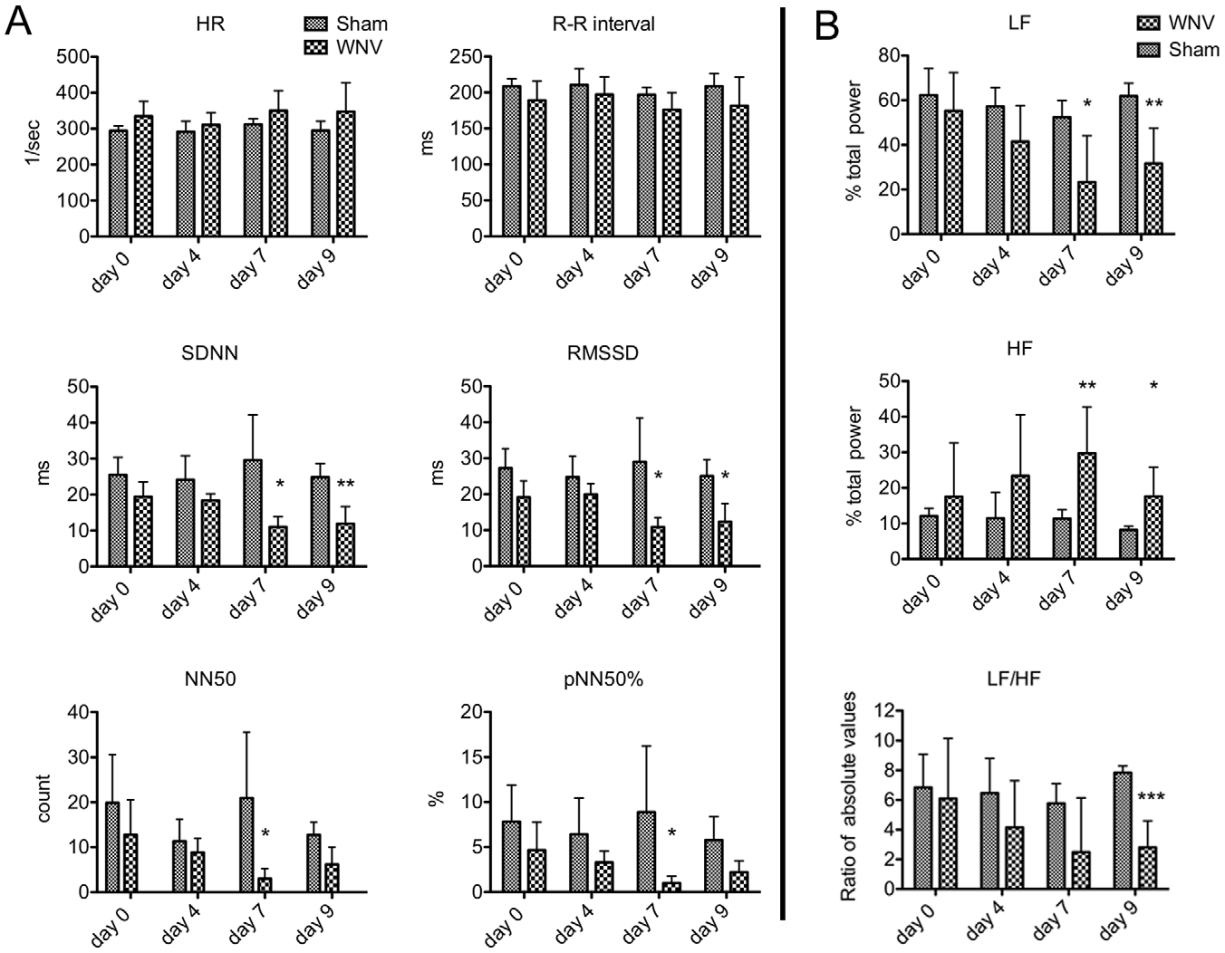
Heart rate variability (HRV) in alert WNV-infected hamsters. Hamsters were surgically implanted with telemetry transmitters with electrodes attached to both pectoralis major muscles, and then injected s.c with WNV (n = 6) or sham (n = 6). ECGs were measured continuously over the course of the experiment. Data were extracted at 0, 4, 7, and 9 days after viral challenge to calculate changes in HRV evaluated in both the A) time domain and B) frequency domain. A) Time domain calculations: R-R intervals (RR); standard deviation of normal RR (SDNN), root mean square of the differences between consecutive RR (RMSSD); number of successive RR interval pairs that differ more than 50 ms (NN50), and NN50 divided by the total number of RR intervals (pNN50) ± SD. B) Frequency domain calculations: Mean low frequency (LF) % total power, high frequency (HF) % total power; and LF/HF ratio of the absolute values ± SD. (*P≤0.05, **P≤0.01, ***P≤0.001 using two-way analysis of variance with the Bonferroni post-test to compared groups at each day or t tests comparing WNV- and sham-data on the same day.)

To investigate the role of the autonomic nervous system on WNV-induced heart dysfunctions, we measured the HRV, which is the time interval variability between heart beats (R-R interval) commonly used as a marker for autonomic function in humans [30] and rodents [31]. HRV is a good marker for autonomic function, because the autonomic nervous system is coupled between the respiratory rate and the heart rate [32]. This cardiorespiratory coupling results in higher variability between of the R-R intervals, in as much as the R-R interval shortens and lengthens during inspiration and expiration of breathing. Essentially, the higher the HRV, the healthier the autonomic nervous system. To measure the R-R interval and HRV, electrocardiographs were obtained by radiotelemetry for up to 9 days after infection. To better interpret these data and their biological significance, the HRV was represented as time domain calculations based on the time of each beat: mean R-R interval (RR), standard deviation of normal R-R intervals (SDNN), root mean square of the differences between consecutive RR intervals (RMSSD), number of successive RR interval pairs that differ more than 50 ms (NN50), and NN50 divided by the total number of RR intervals (pNN50). The mean heart rate (HR) or mean R-R interval data (Figure 5A, top row left) did not reveal any statistical differences between the conscious sham- and WNV-infected animals. However, when the variability (SDNN, RMSSD, NN50, pNN50%) was determined, the HRV was significantly diminished by day 7 in WNV-infected hamsters in all time domain measurements (Figure 5A, middle and bottom rows).

As a result of the cardiorespiratory neurological coupling, the respiratory rate gives rise to waves in the heart rate that is mediated primarily via the parasympathetic autonomic nervous system. These wave patterns at a high frequency (HF) of 1.2–3.6 Hz can be detected by performing Fourier transformation of the R-R intervals. The lag in the baroceptor feedback loop associated with Mayer waves of blood pressure may also contribute to low frequency (LF) patterns (0.32–1.2 Hz) in heart rate. In summary, the HF 1.2–3.6 Hz-wave patterns reflect the influence of the parasympathetic system, and the LF patterns reflect the sympathetic system with some influence from the parasympathetic system. Therefore, we analyzed the frequency domain data since they can provide insights into the involvement of sympathetic or parasympathetic functions [33]. The LF % power was significantly reduced in WNV-infected hamsters beginning on day 7 (P≤0.05) and continuing to day 9 (P≤0.01) (Figure 5B, top). The LF/HF ratio of absolute values also decreased over time, with the data on day 9 (P≤0.0) being statistically significant (Figure 5B, bottom). Conversely, the HF % power was increased over time, with the data on days 7 (P≤0.01) and 9 (P≤0.05) being statistically significant (Figure 5B, middle). These data indicate that autonomic function is affected by WNV infection in hamsters.

**Figure 6:**
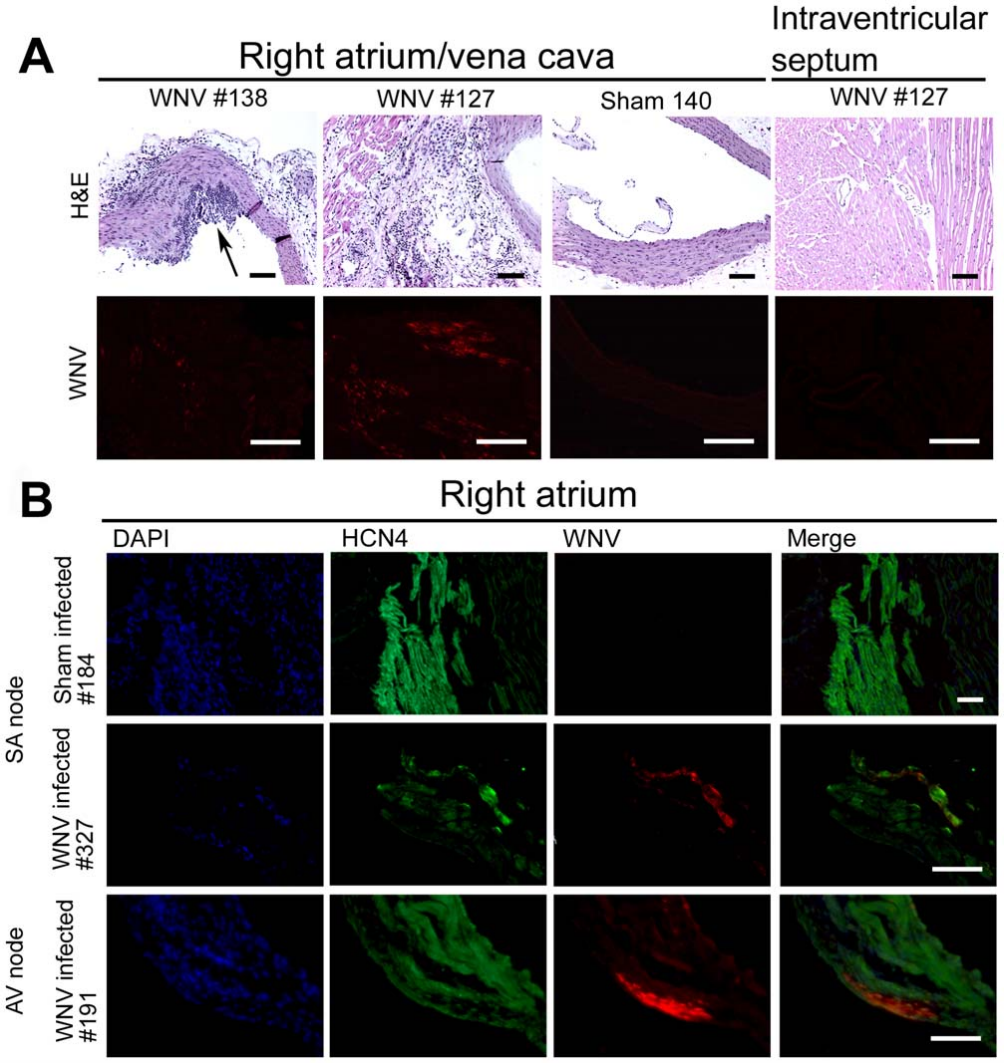
WNV-immunostaining and histopathological immunostaining of the heart in WNV-infected hamsters. Hamsters were injected s.c. with WNV or sham, and necropsied 9 days later. The heart (right atrium, left atrium, right ventricle, and left ventricle) was sectioned for A) H&E staining of the vena cava of the right atrium (A, upper panel), and WNV envelope staining of the vena cava (A, lower panel). Histopathology was only observed in the right atrium, not in other portions of the heart, e.g. intraventricular septum (A, right in lower panel). Histopathological lesions (arrows). B) Immunofluorescent staining of WNV envelope and HCN4 of SA and AV nodes in the right atrium. Bar scale 100 µm.

Arteritis in the vena cava of the heart was identified in all seven of the WNV-infected hamsters evaluated at day 9, but not in any of the six sham-infected hamsters (Figure 6A, arrows). Remarkably, the arteritis was restricted to the right atrium only and was not present in other chambers or tissues of the heart. Endarteritis was identified with moderate to large numbers of luminally marginated neutrophils and lymphocytes. The walls of all affected arteries had multifocal aggregates of necrotic cellular debris and were infiltrated by neutrophils.

We investigated the possibility that WNV might infect cells in the SA and AV nodes of the heart. We employed an antibody specific for potassium/sodium hyperpolarization-activated cyclic nucleotide-gated channel 4 (HCN4) as a marker for SA and AV cells [34] (Figure 6B). Cells stained with WNV were located among cells with the HCN4 marker in the SA and AV nodes in all of the four WNV-infected hamsters assayed. Remarkably like the arteritis, WNV-immunostaining was restricted to the right atrium, and was not detected in other areas of the heart.

The spinal cords and brain stems of the hamsters of this study also contained histopathology and WNV infected neurons (data not shown) as described in prior publications [20], [22], [23], [27], [28], [35].

## Discussion

The contribution of this study is the discovery that WNV infection of rodents can lead to autonomic dysfunction: i.e., reduced HRV, and reduced EMG amplitudes of the GI tract, which is consistent with autonomic dysfunction as evidenced by suppression of the diaphragmatic EMG as previously described [23]. Suppression of the GI EMGs probably led to reduced GI motility as identified by distension of the intestine and retention of phenol red dye. Added evidence of autonomic dysfunction was the reduced HRV, a clinical marker for autonomic dysfunction [36].

Data from this study provide evidence of the existence of WNV-induced autonomic dysfunction, wherein autonomic dysfunction is defined as the dysfunction of neurological control of visceral functions independent of conscious control. Some neurons of the myenteric plexis of the intestine were infected with WNV as evidenced by co-staining for WNV and NSE. The same animals also possessed infected neurons in the brain stem, and prior infected animals possessed infected motor neurons in the spinal cord [22], which suggested that the cause of GI dysfunction was possibly neurologically caused. Peritonitis was present in infected animals compared to no histopathology in the uninfected animals, which suggested that peritonitis may have been the direct or indirect cause of GI dysfunction. The fact that the EMGs, however, were suppressed in the intestines of infected animals suggests that electrophysiological suppression and neurological dysfunction were the cause of GI dysfunction. Therefore, peritonitis may have been the result of GI stasis rather than the cause.

The data of HRV, a well accepted and widely used indicator of autonomic function [36], also indicate a reduction in autonomic functions as evidenced by the reduction of time domain calculations (SDNN, RMSSD, NN50, pNN50%) at successive days after infection. Frequency domain calculations revealed patterns of reduced LF power and LF/HF ratio, and elevated HF power over time. Since HF power is reflective of parasympathetic function and LF power is reflective of sympathetic combined with some parasympathetic functions [32], [37] the reduction in the ratio of LF/HF sympathovagal balance in WNV-infected animals over the course of disease favored a loss of sympathetic more than parasympathetic function [38]. Inasmuch as the sympathetic autonomic function is controlled through the baroceptor and cardiorespiratory reflexes involving the sympathetic ganglia adjacent to the spinal cord, a loss of sympathetic function may be due to infection of motor neurons in the spinal cord [22], [28] relaying to the sympathetic ganglia. Since the medulla oblongata controls cardiorespiratory coupling for coordination between the cardiac and respiratory systems, future studies should investigate the effect of disease in these infected tissues on cardiorespiratory coupling. Since reduced HRV is associated with increased risk of cardiac death [39], and cardiac events such as arrhythmia in patients without coronary heart disease or congestive heart failure [40], [41], this hamster model should be employed to evaluate the effect of WNV infection on heart failure and mortality.

The affect of WNV infected cells and associated histopathology in the right atrium of the heart on the overall health of the hamsters awaits further investigation, but data from this study and other studies may provide some preliminary answers as to the clinical implications. We have previously demonstrated that WNV can spread by retrograde or anterograde transport along axons [27], [28]; therefore the presence of WNV and its associated pathology only in the right atrium may have been due to spreading of the virus along the vagus nerve from the brain stem where robust infection has previously been identified in rodents [23], [38], [42], [43]. To provide preliminary data as to the physiological effects of right atrium involvement, ECGs were recorded and analyzed using telemetry chips. Infection of cells or histopathology in the right atrium did not measurably affect the P wave duration, P-R interval (data not shown). However, skipped beats reflective of AV blockage, and inverted P waves were observed in 4 of 7 WNV-infected sleeping hamsters; no abnormalities were observed in 0 of 7 sham-infected sleeping hamsters (data not shown). Therefore, the infection and associated pathology in the right atrium may have contributed to ECG abnormalities. Alternatively, the ECG abnormalities may have been caused by brainstem infection, because serum creatine kinase activity was not elevated to pathological levels in infected hamsters (data not shown). The lack of overt myocarditis, evidence for skipped beats (arrhythmia), and gastrointestinal involvement may reflect the human disease in as much as cardiomyopathy is only rarely observed [44], [45], whereas GI distress is a predominant symptom of WNV infection in hospitalized patients of one study [4], and cardiac arrhythmia occurred in all groups of patients with fever, meningitis, or encephalitis in a Colorado study [9]. The implications of these cardiac data in the hamsters and in the human disease await further investigations.

The spectrum of symptoms of human West Nile neurological disease includes respiratory distress [16], diaphragmatic paralysis [2], gastrointestinal involvement, urinary retention [17], bladder dysfunction [16], fatigue [46], and cardiac arrhythmia [9]. Since all of these physiological functions depend on the autonomic nervous system, the rodent data presented herein may be analogous to conditions leading to many of the symptoms of WNV-infected human patients.

## Materials and Methods

### Ethics Statement

This study was conducted in accordance with the approval of the Institutional Animal Care and Use Committee of Utah State University (IACUC approval #1079 and #1488; WNV APHIS Permit # 47210). The work was done in the AAALAC-accredited Laboratory Animal Research Center of Utah State University and in accordance with the National Institutes of Health Guide for the Care and Use of Laboratory Animals.

### Animals and Viruses

Adult female Syrian golden hamsters (>7 weeks old) were used (Charles River Laboratories). Animals were randomized to treatment groups by blindly selecting hamsters from a common container. This study was conducted in accordance with the approval of the Institutional Animal Care and Use Committee of Utah State University. A New York WNV isolate (NY WNV) [47], [48] from crow brain was used. The virus propagated in MA-104 cells and diluted in minimal essential medium (MEM) immediately prior to subcutaneous (s.c.) injection in the groin area [23], [38], [43]. Hamsters and mice were injected with a volume of 0.1 mL containing 5.7×10^7^ and 5.7×10^5^ pfu, respectively.

### Gastrointestinal transit assay

On day 9 after injection, a volume of 1.5 mL of phenol red (0.5 mg/mL in 5% glucose) was administered to fasting (24 hr) hamsters (5 sham-injected and 7 WNV-injected) by oral gavage [49]. Ten min later, the stomach was removed. The tissue volume was determined by measuring the displacement of 10 mL of 0.1 N NaOH, after which the tissue was homogenized. After 20 min at room temperature, the supernatants were collected by centrifuged for 10 min at 2,800 rpm. After precipitation of proteins with trichloroacetic acid (20%), samples were centrifuged. The absorbance of 3 mL of supernatant added to 4 mL of 0.5 N NaOH was read at 560 nm and expressed as the relative concentrations of phenol red in each stomach.

### Nerve conduction velocity (NCV)

In 8 sham- and 8 WNV-injected hamsters, motor NCV was determined from the surface distance (millimeters) from the proximal and to the distal stimulating electrodes divided by the time (milliseconds) required for the impulse to travel between these points (velocity = distance/time). To validate the NCV, 5 animals untreated, and 5 untreated animals with lidocaine intramuscular injection add the experiment. Sensory nerve conduction velocity, uncontained compound muscle action potential, was obtained by stimulating a mixed nerve and recorded from a purely sensory branch. The calculation was the distance divided by onset of latency [50], [51].

The temperature of isoflurane-anesthetized hamsters on a heating pad was regulated 37±0.5°C with an automatically regulated rectal thermometer controller (TCAT-2, Physitemp Instruments, Inc., Clifton, NJ). For the sciatic-tibial motor nerve, the left hind limb and dorsal thigh were sheared and scrubbed with iodine solution and ethanol, wherein the proximal and distal stimulate acupuncture needles (36, 0.2×13 mm, Tai Chi, Lhass Medical, Inc., Accord, MA) were inserted into gluteus maximus muscle at the sciatic notch and the tibial nerve posterior to the medial malleolus. The recording electrode was inserted in the lateral plantar. The supramaximal stimulus with a pulse of 0.1 ms duration was delivered from an Isostim stimulator/isolator A320 (World Precision Instruments, Sarasota, FL). The reference electrode was inserted into the center of foot. The evoked muscle action potentials were amplified. The LabVIEW 8.2 virtual instrument program (National Instruments Corporation, Austin, TX) interfaced with the computer was used for recording and measuring conduction time. The distance between two stimulating electrodes was measured with a ruler. Antidromic median sensory nerve conduction velocity was performed similarly by inserting the stimulation electrode into carpal tunnel and the recording electrode on the lateral subcutaneous index digit [22], [52], [53].

### Electromyography (EMG) of the intestine

Based on previous procedures [54], [55] a small incision was made through the abdominal musculature below the right costal margin to expose the duodenum. A pair of 40-gauge Teflon™-insulated single-stranded stainless steel wires (AS765, Cooner Wire Specialty Wire & Cable, Chatsworth, CA) was implanted in the duodenum. The first electrode was placed 4–5 mm posterior to the pylorus and the second was implant 4–5 mm posterior to the first electrode. This was accomplished by inserting into the muscle a 26-gauge needle containing the electrode wire with a 1 mm bare tip bent into a hook. When the needle was removed, the electrode remained in the muscle. The differential electrode leads were routed subcutaneously to exit on the dorsal surface of the back. After surgery, hamsters were treated with an antibiotic (Baytril) and buprenophine. On day 3 after surgery, 5 hamsters were injected each with WNV and sham. On 9 day after viral challenge, hamsters were fasted for 15 hours before recording EMG of duodenum. Under isoflurane anesthesia, leads were connected to a differential amplifier (DAM50, World Precision Instruments, Sarasota, FL) set at 1K-gain and the band-pass filter between 300 Hz and 1 kHz. EMG recordings were monitored on a digital storage oscilloscope (2542BK BK Precision Electronic Test Instruments, Yorba Linda, CA). Recordings were made consecutively 2 min before and 2 min after 5% glucose oral gavage at the end of a phase III migrating myoelectric complex. The instrument calculated the root mean square (RMS) of the amplitude of the waveform for these two 2-min recordings.

### Monitoring heart rate variability (HRV) by radio telemetry

Based on previous procedures [31], [56], hamsters were anaesthetized with a ketamine-xylazine cocktail (100 mg/mL ketamine & 5 mg/mL xylazine) at 0.1 mL per 100 grams of animal weight, and administered buprenorphine at 0.1 mg/kg for pain control. Anesthesia was kept constant with 2% isoflurane carried in 1 L/min of oxygen for the duration of the procedure. After the hamsters were prepared for surgery, a midline dorsal incision was made along spine and a subcutaneous pocket was made to house the telemetric device (TA10EEAT-F20, Data Science International). Two recording-leads were subcutaneously tunneled toward the left and right clavicular region where the tips of leads were sutured to the pectoral muscles. Six animals were then s.c. injected with WNV or sham each. The DSI ETA-F20 transmitter transmits temperature, heart rate, and animal activity as an analog radio signal, with a digital signature. The duration of each reading was 60 s, and 1–2 readings were obtained at midnight (±2 hr) and at noon (±2 hr). The values for one complete day were averaged. The receivers, under the cages, were connected to a data acquisition matrix hard-wired to a PC-based computer running Dataquest A.R.T. Silver System software. The tabulated data were then exported to a spreadsheet. Graphical analysis was performed using Prism (GraphPad Software, Inc.).

To interpret the HRV data and the biological significance, frequency domain was analyzed with the power spectral densities (PSD) of the R-R interval time series based on the fast Fourier transform. The PSD was analyzed by calculating powers and peak frequencies for different frequency bands using Kubios HRV software (version 2.0, University of Kuopio, Kuopio, Finland) [57]. Cut-off frequencies developed specifically for hamsters were 0.32–1.2 Hz for low frequencies (LF) and 1.2–3.6 Hz for high frequencies (HF) [31]. For spectrum estimation, the interpolation of RR series was 12 Hz, the points in frequency-domain were 512 points/Hz, and the window width was 256 second and 50% overlap.

For the time domains based on the time of each beat as measured from the ECG R peak, the mean R-R interval (RR), standard deviation of normal R-R intervals (SDNN), root mean square of the differences between consecutive RR intervals (RMSSD), number of successive RR interval pairs that differ more than 50 ms (NN50), and NN50 divided by the total number of RR intervals (pNN50) were calculated for each hamster.

### GI histology

The light microscopy and fluorescence microscopy were described elsewhere [22], [58], in which we performed relevant controls such as absence of the primary antibody, negative controls from uninfected animals, positive controls from kidneys of WNV-infected animals and WNV-infected Vero cells, and confirmation of antibody specificity using different staining protocols. Histopathological lesions were identified using paraffinized sections (5 µm) with hematoxylin/eosin (H&E) staining.

To prepare myenteric plexuses for immunohistochemistry at day 8, 4 sham- and 4 WNV-injected hamsters were perfused with PBS followed by 4% paraformaldehyde from which the duodenum, jejunum, ileum and colon were removed. Samples were longitudinally opened and pinned flat in a Sylgard dish, the mucosa and the submucosa were gently scraped off, and the circular layer muscle was removed under a dissecting microscope. Longitudinal muscles with myenteric plexuses were mounted on pre-coated poly-l-lysine glass slides. Samples were dried at room temperature [59].

To identify WNV-infected cells in the vicinity of synaptic terminals in the myenteric ganglia using fluorescence microscopy, tissues were double-stained with primary rabbit monoclonal anti-synaptophysin antibody (Chemicon, Temecula, CA) [60] and mouse monoclonal WNV env-specific antibody 7H2 (BioReliance, Invitrogen Bioservices, Rockville, MD). To identify neurons specifically infected with WNV in myenteric ganglia, tissues were double-stained with neuron-specific enolase (NSE) (Chemicon,Temecula, CA) and the 7H2 antibody. Secondary antibodies for both assays were Alexa-fluor® 488 goat anti-rabbit IgG and Alexa Fluor 586 goat anti-mouse IgG (Molecular Probes, Eugene, OR) [61].

### Heart and medulla oblongata histology

To dissect the SA node, the right atrium was opened along the caval axis to expose the crista-terminalis and SA node [34] in 2 sham- and 4 WNV-injected hamsters. The AV node was dissected using the landmarks of the triangle of Koch, septal tricuspid valve, and the tendon of Todaro [62], [63]. Cryostat sections (10 µm) were used for immunohistochemical double-staining with rabbit anti-HCN4 antibody (US biological, Swampscott, MA) and mouse monoclonal WNV env-specific 7H2 antibody. To correlate the pathological lesions with WNV in the heart, serial sections of the right atrium were stained for WNV using immunofluorescence and H&E. For the WNV immunofluorescent staining, paraffin sections (5 µm) were deparaffinized, rehydrated, antigen retrieved with DakoCytomation target retrieval solution in pressure cooker, and incubated with the 7H2 primary antibody. A board-certified veterinary pathologist blinded to the identification of the samples examined the H&E stained sections.

## References

[1] Sejvar JJ, Bode AV, Marfin AA, Campbell GL, Ewing D (2005). West Nile virus-associated flaccid paralysis.. Emerg Infect Dis.

[2] Betensley AD, Jaffery SH, Collins H, Sripathi N, Alabi F (2004). Bilateral diaphragmatic paralysis and related respiratory complications in a patient with West Nile virus infection.. Thorax.

[3] Sejvar JJ, Bode AV, Marfin AA, Campbell GL, Pape J (2006). West Nile Virus-associated flaccid paralysis outcome.. Emerg Infect Dis.

[4] Weiss D, Carr D, Kellachan J, Tan C, Phillips M (2001). Clinical findings of West Nile virus infection in hospitalized patients, New York and New Jersey, 2000.. Emerg Infect Dis.

[5] Herlocher ML, Truscon R, Elias S, Yen HL, Roberts NA (2004). Influenza viruses resistant to the antiviral drug oseltamivir: transmission studies in ferrets.. J Infect Dis.

[6] Jeha LE, Sila CA, Lederman RJ, Prayson RA, Isada CM (2003). West Nile virus infection: a new acute paralytic illness.. Neurology.

[7] Lindsey NP, Staples JE, Lehman JA, Fischer M (2010). Surveillance for human West Nile virus disease - United States, 1999-2008.. MMWR Surveill Summ.

[8] Pergam SA, DeLong CE, Echevarria L, Scully G, Goade DE (2006). Myocarditis in West Nile Virus infection.. Am J Trop Med Hyg.

[9] Bode AV, Sejvar JJ, Pape WJ, Campbell GL, Marfin AA (2006). West Nile virus disease: a descriptive study of 228 patients hospitalized in a 4-county region of Colorado in 2003.. Clin Infect Dis.

[10] Omalu BI, Shakir AA, Wang G, Lipkin WI, Wiley CA (2003). Fatal fulminant pan-meningo-polioencephalitis due to West Nile virus.. Brain Pathol.

[11] Lichtensteiger CA, Heinz-Taheny K, Osborne TS, Novak RJ, Lewis BA (2003). West Nile virus encephalitis and myocarditis in wolf and dog.. Emerg Infect Dis.

[12] Gibbs SE, Ellis AE, Mead DG, Allison AB, Moulton JK (2005). West Nile virus detection in the organs of naturally infected blue jays (Cyanocitta cristata).. J Wildl Dis.

[13] Harrison AK, Murphy FA, Gardner JJ (1982). Visceral target organs in systemic St. Louis encephalitis virus infection of hamsters.. Exp Mol Pathol.

[14] Obeyesekere I, Hermon Y (1973). Arbovirus heart disease: myocarditis and cardiomyopathy following dengue and chikungunya fever—a follow-up study.. Am Heart J.

[15] De Brito T, Siqueira SA, Santos RT, Nassar ES, Coimbra TL (1992). Human fatal yellow fever. Immunohistochemical detection of viral antigens in the liver, kidney and heart.. Pathol Res Pract.

[16] Saad M, Youssef S, Kirschke D, Shubair M, Haddadin D (2005). Acute flaccid paralysis: the spectrum of a newly recognized complication of West Nile virus infection.. J Infect.

[17] Shpall AI, Varpetian A, Ginsberg DA (2003). Urinary retention in a patient with West Nile virus.. Urology.

[18] Abroug F, Ouanes-Besbes L, Ouanes I, Nciri N, Dachraoui F (2006). Adrenal insufficiency in severe West Nile Virus infection.. Intensive Care Med.

[19] Xiao SY, Guzman H, Zhang H, Travassos da Rosa AP, Tesh RB (2001). West Nile virus infection in the golden hamster (Mesocricetus auratus): a model for West Nile encephalitis.. Emerg Infect Dis.

[20] Morrey JD, Siddharthan V, Wang H, Hall JO, Skirpstunas RT (2008). West Nile virus-induced acute flaccid paralysis is prevented by monoclonal antibody treatment when administered after infection of spinal cord neurons.. J Neurovirol.

[21] Morrey JD, Day CW, Julander JG, Olsen AL, Sidwell RW (2004). Modeling hamsters for evaluating West Nile virus therapies.. Antiviral Res.

[22] Siddharthan V, Wang H, Motter NE, Hall JO, Skinner RD (2009). Persistent West Nile virus associated with a neurological sequela in hamsters identified by motor unit number estimation.. J Virol.

[23] Morrey JD, Siddharthan V, Wang H, Hall JO, Motter NE (2010). Neurological suppression of diaphragm electromyographs in hamsters infected with West Nile virus.. J Neurovirol.

[24] Tesh RB, Siirin M, Guzman H, Travassos da Rosa AP, Wu X (2005). Persistent West Nile virus infection in the golden hamster: studies on its mechanism and possible implications for other flavivirus infections.. J Infect Dis.

[25] Tonry JH, Xiao SY, Siirin M, Chen H, da Rosa AP (2005). Persistent shedding of West Nile virus in urine of experimentally infected hamsters.. Am J Trop Med Hyg.

[26] Doron SI, Dashe JF, Adelman LS, Brown WF, Werner BG (2003). Histopathologically proven poliomyelitis with quadriplegia and loss of brainstem function due to West Nile virus infection.. Clin Infect Dis.

[27] Samuel MA, Wang H, Siddharthan V, Morrey JD, Diamond MS (2007). Axonal transport mediates West Nile virus entry into the central nervous system and induces acute flaccid paralysis.. Proc Natl Acad Sci U S A.

[28] Wang H, Siddharthan V, Hall JO, Morrey JD (2009). West Nile virus preferentially transports along motor neuron axons after sciatic nerve injection of hamsters.. J Neurovirol.

[29] McMahon HT, Bolshakov VY, Janz R, Hammer RE, Siegelbaum SA (1996). Synaptophysin, a major synaptic vesicle protein, is not essential for neurotransmitter release.. Proc Natl Acad Sci U S A.

[30] Park SK, Auchincloss AH, O'Neill MS, Prineas R, Correa JC (2010). Particulate air pollution, metabolic syndrome, and heart rate variability: the multi-ethnic study of atherosclerosis (MESA).. Environ Health Perspect.

[31] Mongue-Din H, Salmon A, Fiszman MY, Fromes Y (2009). Periodic variation in R-R intervals and cardiovascular autonomic regulation in young adult Syrian hamsters.. Am J Physiol Regul Integr Comp Physiol.

[32] Ori Z, Monir G, Weiss J, Sayhouni X, Singer DH (1992). Heart rate variability. Frequency domain analysis.. Cardiol Clin.

[33] Bilchick KC, Berger RD (2006). Heart rate variability.. J Cardiovasc Electrophysiol.

[34] Viswanathan S, Burch JB, Fishman GI, Moskowitz IP, Benson DW (2007). Characterization of sinoatrial node in four conduction system marker mice.. J Mol Cell Cardiol.

[35] Morrey JD, Siddharthan V, Olsen AL, Roper GY, Wang HC (2006). Humanized monoclonal antibody against West Nile virus E protein administered after neuronal infection protects against lethal encephalitis in hamsters.. Journal of Infectious Disease.

[36] Malik M, Bigger JT, Camm AJ, Kleiger RE, Malliani A, Moss AJ, Schwartz PJ (1996). Heart rate variability. Standards of measurement, physiological interpretation, and clinical use. Task Force of the European Society of Cardiology and the North American Society of Pacing and Electrophysiology.. Eur Heart J.

[37] Zapanta L (2005). Heart rate variability in mice with coronary heart disease [Master of Science]: Massachusetts Institute of Technology.

[38] Morrey JD, Siddharthan V, Olsen AL, Wang H, Julander JG (2007). Defining limits of treatment with humanized neutralizing monoclonal antibody for West Nile virus neurological infection in a hamster model.. Antimicrob Agents Chemother.

[39] Stein PK, Domitrovich PP, Huikuri HV, Kleiger RE (2005). Traditional and nonlinear heart rate variability are each independently associated with mortality after myocardial infarction.. J Cardiovasc Electrophysiol.

[40] Tsuji H, Larson MG, Venditti FJ, Manders ES, Evans JC (1996). Impact of reduced heart rate variability on risk for cardiac events. The Framingham Heart Study.. Circulation.

[41] Huikuri HV, Valkama JO, Airaksinen KE, Seppanen T, Kessler KM (1993). Frequency domain measures of heart rate variability before the onset of nonsustained and sustained ventricular tachycardia in patients with coronary artery disease.. Circulation.

[42] Hunsperger EA, Roehrig JT (2006). Temporal analyses of the neuropathogenesis of a West Nile virus infection in mice.. J Neurovirol.

[43] Morrey JD, Siddharthan V, Olsen AL, Roper GY, Wang H (2006). Humanized monoclonal antibody against West Nile virus envelope protein administered after neuronal infection protects against lethal encephalitis in hamsters.. J Infect Dis.

[44] Khouzam RN (2009). Significant cardiomyopathy secondary to West Nile virus infection.. South Med J.

[45] Kushawaha A, Jadonath S, Mobarakai N (2009). West nile virus myocarditis causing a fatal arrhythmia: a case report.. Cases J.

[46] Leis AA, Stokic DS (2005). Neuromuscular Manifestations of Human West Nile Virus Infection.. Curr Treat Options Neurol.

[47] Lanciotti RS, Ebel GD, Deubel V, Kerst AJ, Murri S (2002). Complete genome sequences and phylogenetic analysis of West Nile virus strains isolated from the United States, Europe, and the Middle East.. Virology.

[48] Lanciotti RS, Kerst AJ (2001). Nucleic acid sequence-based amplification assays for rapid detection of West Nile and St. Louis encephalitis viruses.. J Clin Microbiol.

[49] Kabatas S, Yu D, He XD, Thatte HS, Benedict D (2008). Neural and anatomical abnormalities of the gastrointestinal system resulting from contusion spinal cord injury.. Neuroscience.

[50] Franssen H, van den Bergh PY (2006). Nerve conduction studies in polyneuropathy: practical physiology and patterns of abnormality.. Acta Neurol Belg.

[51] Mayer RF (1966). Peripheral nerve conduction in alcoholics: Studies of th effects of acute and chronic intoxication.. Psychosomatic Medicine.

[52] Thomas PK, Jefferys JG, Sharma AK, Bajada S (1981). Nerve conduction velocity in experimental diabetes in the rat and rabbit.. J Neurol Neurosurg Psychiatry.

[53] Schmelzer JD, Zochodne DW, Low PA (1989). Ischemic and reperfusion injury of rat peripheral nerve.. Proc Natl Acad Sci U S A.

[54] Rodriguez-Membrilla A, Martinez V, Vergara P (1995). Peripheral and central cholecystokinin receptors regulate postprandial intestinal motility in the rat.. J Pharmacol Exp Ther.

[55] Fioramonti J, Fargeas MJ, Bertrand V, Pradayrol L, Bueno L (1994). Induction of postprandial intestinal motility and release of cholecystokinin by polyamines in rats.. Am J Physiol.

[56] Sgoifo A, Stilli D, Medici D, Gallo P, Aimi B (1996). Electrode positioning for reliable telemetry ECG recordings during social stress in unrestrained rats.. Physiol Behav.

[57] Niskanen JP, Tarvainen MP, Ranta-Aho PO, Karjalainen PA (2004). Software for advanced HRV analysis.. Comput Methods Programs Biomed.

[58] Morrey JD, Siddharthan V, Wang H, Hall JO, Skirpstunas RT (2008). West Nile virus-induced acute flaccid paralysis is prevented by monoclonal antibody treatment even after infection of spinal cord neurons.. Journal of Neurovirology.

[59] Buttow NC, Santin M, Macedo LC, Neres Teixeira AC, Novakowski GC (2004). Study of the myenteric and submucous plexuses after BAC treatment in the intestine of rats.. Biocell.

[60] Vernino S, Low PA, Lennon VA (2003). Experimental autoimmune autonomic neuropathy.. J Neurophysiol.

[61] Sarnelli G, De Giorgio R, Gentile F, Cali G, Grandone I (2009). Myenteric neuronal loss in rats with experimental colitis: role of tissue transglutaminase-induced apoptosis.. Dig Liver Dis.

[62] Petrecca K, Amellal F, Laird DW, Cohen SA, Shrier A (1997). Sodium channel distribution within the rabbit atrioventricular node as analysed by confocal microscopy.. J Physiol.

[63] Yoo S, Dobrzynski H, Fedorov VV, Xu SZ, Yamanushi TT (2006). Localization of Na+ channel isoforms at the atrioventricular junction and atrioventricular node in the rat.. Circulation.

